# Sydney Reporting System for Lymph Node Fine-Needle Aspiration and Malignancy Risk Stratification: Is It of Clinical Value?

**DOI:** 10.3390/diagnostics14161801

**Published:** 2024-08-17

**Authors:** Doaa Alqaidy, Hind Althomali, Amal Almaghrabi

**Affiliations:** 1 Department of Pathology, King Abdulaziz University, Jeddah 22252, Saudi Arabia; 2 Hematology Research Unit, King Fahd Medical Research Center, King Abdulaziz University, Jeddah 22252, Saudi Arabia; 3 Department of Pathology, King Abdulaziz University Hospital, Jeddah 22252, Saudi Arabia

**Keywords:** lymph node cytology, fine-needle aspiration, Sydney reporting system, risk of malignancy

## Abstract

Lymphadenopathy is a common presentation of both reactive and malignant diseases, and lymph node fine-needle aspiration cytology (LN-FNAC) is an effective and inexpensive screening method. It can prevent unnecessary invasive surgery and excisional biopsy, especially in benign cases. Unfortunately, the lack of universally accepted terminology for reporting results has hindered its widespread support. The Sydney system proposal for lymph node cytopathology categorization and reporting introduced five diagnostic categories to address the lack of universally accepted terminology for reporting results in lymphadenopathy. Our study analyzed 188 lymph node fine-needle cytology (FNC) samples from King Abdulaziz University Hospital, Saudi Arabia, examining clinical follow-up data, pathology records, patient information, and final diagnosis from January 2019 to December 2022. Most specimens were from axillary lymph nodes, with 99.5% tissue correlation. The Sydney system category classification identified 56.9% of cases as malignant, while 26.1% were benign. The final surgical specimen diagnosis revealed a higher percentage of malignant diagnoses, with the highest risk of malignancy (ROM) in malignant/category V. In conclusion, our study demonstrates that LN-FNAC offers high diagnostic accuracy for lymph node (LN) aspirates, with the Sydney approach potentially aiding risk stratification and achieving consistency in cytologic diagnosis, but further multi-centric research is needed.

## 1. Introduction

Lymphadenopathy is a prevalent manifestation of both reactive and malignant diseases. When it comes to screening for and, to a lesser degree, diagnosing lymphadenopathy, fine-needle aspiration (FNA) is still an effective and inexpensive option [1,2,3]. While tissue biopsies are often necessary for lymphoma diagnosis, FNA is also a useful method for obtaining material for further ancillary tests such as flow cytometry, microbiological cultures, and molecular analysis [4]. Although it remains a subject of debate, fine-needle aspiration is a valuable screening technique that can assist prevent unnecessarily invasive surgery and excisional biopsy, particularly in instances with a low likelihood of lymphoma in benign lymphadenopathy cases [2].

Moreover, this procedure is straightforward and can be performed at modest healthcare facilities with little resources. Even a general practitioner can perform it with the aid of ultrasound guidance [5]. It also remains a valuable tool in situations where surgical biopsies cannot be conducted due to the patient’s severe illness or the potential for secondary surgery-related complications. Furthermore, fine-needle aspiration is an invaluable tool for evaluating and directing the choice of neo-adjuvant treatment in patients with suspected node-positive breast carcinoma [6].

One of the biggest obstacles in lymph node cytology is the lack of a universally accepted terminology for reporting lymph node FNA cytology results [7]. This is among the reasons that lymph node FNA has not gained widespread support from medical practitioners and oncologists, despite its minimum invasiveness, low cost, and speed [1,3,8,9].

At the 20th International Congress of Cytology in Sydney in May 2019, an expert group released the Sydney system proposal for lymph node cytopathology categorization and reporting [10], with the introduction of the following five diagnostic categories: category I/L1 represents inadequate or nondiagnostic results, category II/L2 indicates benign findings, category III/L3 refers to atypical cells of unknown significance or atypical lymphoid cells of uncertain significance, category IV/L4 suggests suspicious results, and category V/L5 represents malignant results [11,12,13].

Nevertheless, there is a lack of data in the literature and the Sydney method has yet to be utilized sufficiently. This study aims to fill this knowledge gap by comparing the ROM for each diagnostic category, the accuracy of the diagnosis, and the efficacy of the Sydney reporting system for lymph node FNA [8,10].

## 2. Materials and Methods

### 2.1. Study Design

This study conducted a retrospective analysis on 188 lymph node fine-needle aspiration cytology (FNC) samples received from the Department of Pathology at King Abdulaziz University Hospital (KAUH) in Jeddah, Saudi Arabia. The samples were collected between 1 January 2019 and 31 December 2022. This research includes lymph node aspirates from individuals of all genders and all age groups. Excluded were any lymph node aspirates that did not have a subsequent tissue biopsy. The clinical follow-up data and glass slides of the included cases were examined. The pathology records were obtained and information on the patients’ age, gender, location of lymph nodes, clinical history, further testing, and ultimate diagnosis were documented.

**Figure 1:**
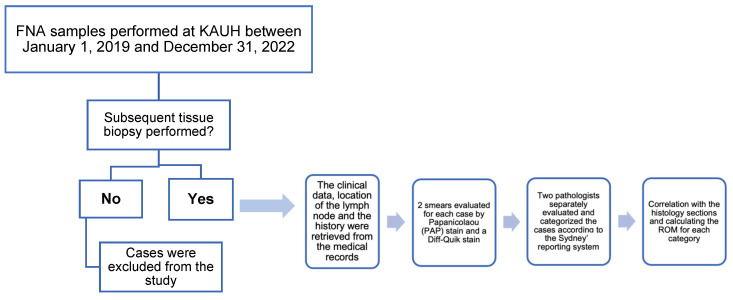
The detailed study design with the inclusion and exclusion criteria used.

Fine-needle aspiration (FNA) was conducted with strict adherence to aseptic measures in all cases. A percutaneous fine-needle aspiration cytology (FNAC) was conducted on the superficial lymph nodes using a 22-gauge hypodermic needle. For deeply located lymph nodes, radiologic guidance, either through ultrasonography or computed tomography, was used. Each case attempted a minimum of 2 passes. For each case, a minimum of 2 smears were made, both air-dried and wet-fixed. Additionally, a Papanicolaou (PAP) stain and a Diff-Quik stain were prepared for each case (Figure 1).

### 2.2. Diagnostic Criteria

Two cytopathologists conducted an independent evaluation and categorization of the smears according to the suggested classification scheme. They were unaware of the final histological diagnosis. All inconsistencies in the categorization were resolved by agreement. The diagnostic criteria for each group are based on the proposed Sydney reporting system and are outlined in provided Table 1.

### 2.3. Histopathological Correlation

The histopathologic diagnoses, where available, were then compared with the cytopathologic diagnoses. The examples that exhibited discordance were examined and the likely causes for the lack of agreement were determined. Both the histology and fine-needle aspiration cytology (FNC) diagnosis of all included cases were found to be consistent and in agreement. The ROM (risk of malignancy) was evaluated for each diagnostic group based on histopathologic correlation and the likelihood of malignant outcomes.

### 2.4. Statistical Analysis

Data were statistically analyzed using the SPSS application version 26. To investigate the association between the variables, the Chi-squared test (χ2) was applied to qualitative data, which were expressed as numbers and percentages. Quantitative data were expressed as mean and standard deviation (Mean ± SD). The diagnostic accuracy of the Sydney system category for the prediction of malignant outcomes was assessed in terms of sensitivity, specificity, and positive and negative predictive values. Sensitivity was defined as the ability of the test to correctly identify those who had the disease (true + ve/true + ve + false – ve). Specificity was defined as the ability of the test to correctly identify those who did not have the disease (True – ve/false + ve + true – ve). The predictive value (+ve) (PPV) was defined as the proportion of individuals screened positive by the test who actually had the disease (PPV) = (true + ve/true + ve + false + ve) and the negative predictive value (-ve) (NPV) was the same for negatives = (true – ve/true – ve + false – ve) [14].

The accuracy rate was the fraction of predictions that the used modality model predicted accurately (accuracy = number of correct predictions/total number of predictions) and it equals the following: (true + ve + true - ve/true + ve + true - ve + false + ve + fale - ve) (1). The true positive cases were those diagnosed as malignant in the final surgical specimen diagnosis and also as class L5: malignant in the Sydney system, and the true negative cases were those diagnosed as benign in the final surgical specimen diagnosis and as class L 1, 2, 3, or 4 in the Sydney system. The false positive cases were those diagnosed as class L5: malignant in the Sydney system but as benign in the final surgical specimen diagnosis. The false negatives were those diagnosed as class L 1, 2, 3, or 4 in the Sydney system but as malignant in the final surgical specimen diagnosis. A *p*-value of less than 0.05. was considered statistically significant.

## 3. Results

Of the studied 188 patients, 58% had an age of more than 50 years with a mean age of 51.22 ± 16.4 years. Of these, 62% were females (Table 2).

As for the specimen source, most of specimens were from the axillary LNs and 99.5% had a tissue correlation. According to the Sydney system, most of the cases (56.9%) were classified as category V (L5): malignant, and 26.1% were classified as category II (L2): benign. After the final surgical specimen diagnosis, 64.4% of specimens were diagnosed as malignant (Table 2).

Table 3 demonstrates that, when comparing the Sydney system category classification with the final surgical specimen diagnosis, the sensitivity of the Sydney system category classification was 78.5% and its specificity was 82%. The PPV was 88.7% and the NPV was 67.9%. The accuracy rate of the Sydney system category classification was 79.7%.

The correlation between the final surgical specimen diagnosis and the Sydney system categories revealed that malignant diagnoses had a significantly higher chance of being classified as L5: malignant in the Sydney system (*p* ≤ 0.05) (Table 4). 

The detailed list of malignant cases in different diagnostic categories and the associated ROM in each category are presented in Table 5. The ROM was lowest (14.2%) for the benign category II (L2) and highest (88.76%) for the malignant category V (L5). The χ2 test revealed that this difference was statistically significant (χ2 = 13.95, *p*-value ≤ 0.001). 

## 4. Discussion

Fine-needle aspiration (FNA) remains an important technique used as a first line diagnostic approach for most lymphadenopathy of unknown etiology [5]. Its broad applicability in the assessment of lymphadenopathy is facilitated by its minimal invasiveness, rapidity, cost-effectiveness, and the ability to provide material for multiple ancillary techniques [4]. Nevertheless, the traditional, currently used method of reporting lymph node smears lacks a consistent diagnostic categorization, a shared language of reporting among cytopathologists, and unambiguous communication to physicians regarding the risk of malignancy and subsequent therapy [3,7,15].

In recent decades, with the introduction of the Bethesda reporting system for cervical cytology [16], the implementation of standardized reporting methods in cytopathology has been seen to decrease intra-observer variability in reporting. It also facilitates communication within the clinical world and helps the transfer of clinically important data in a consistent and replicable way. In addition, it guides clinical teams with regard to management and risk stratification. 

In May 2019, during the International Congress of Cytology in Sydney, a systemic approach was proposed for the classification, reporting, and execution of lymph node cytology [9,10,17,18]. Their main aim was to develop a common language and to help cytopathologists, hematopathologists, physicians, surgeons, and other medical professionals communicate more effectively, establish consensus criteria and a reference framework. Moreover, the system aimed to offer management suggestions associated with reporting categories, which may have involved utilizing clinical and imaging follow-up, supplementary testing, and potential requirement for LN excision. This will ultimately enhance the accuracy of LN-FNAC and raise awareness among clinicians on its diagnostic capabilities [10,17,19].

Before a newly proposed classification system can be recommended for everyday use, it is necessary to establish its validity, repeatability, and clinical value. For this purpose, several research studies have been conducted at various academic institutions throughout various geographical areas. In this series, we demonstrated the Sydney system’s capacity to classify lymph node FNCs into groups with progressively greater ROMs [3,13,20].

In our study, we included a total of 188 cases. The number of cases falling into each category were as follows: L1 (inadequate/nondiagnostic)—6 cases (33.3%), L2 (benign)—49 cases (14.2%), L3 (atypical cells of undetermined significance/atypical lymphoid cells of uncertain significance)—15 cases (66.6%), L4 (suspicious)—11 cases (63.6%), and L5 (malignant)—107 cases (88.7%). Therefore, L5 was the category that was utilized most frequently. Remarkably, the ROM (risk of malignancy) for category V (L5) was very high, totaling 56.1%. The fact that our hospital is a tertiary care facility means that we treat a disproportionately large number of cancer patients, which may explain why our results are so far above the average in many previously published studies.

The ROM for category I (L1) was shown to be rather low, at 33.2%. Of the six cases in this category, we found that most of these smears showed blood and necrosis, which may be related to the expertise of the clinician or the radiologist, as most of the FNAs at our institute are performed by non-cytopathologists. This rate was similar to the results reported by Parikshaa Gupta et al. and much lower than those reported by Elena Vigliar et al., as they reported category I/L1 to be more populated, at 26.5% and 50%, respectively [18,21]. Category II/L2 (benign) category results in our study were comparable to their data, at 14.2%. After further surgical biopsy, 7 of the 49 cases in this group proved to be malignant. Reviewing these cases, we found that, in a small number of cases with Hodgkin lymphoma, the RS cells were spared, and the cytology sample did not accurately represent the disease. Furthermore, three cases comprised metastatic breast ductal carcinoma, in which no carcinoma cells were detected in the cytology specimens and no cell block was conducted. 

Category III (L3), also known as atypia of indeterminate significance, was implemented by the Sydney system to ensure a high accuracy in identifying benign and malignant cases by maintaining high negative and positive predictive values, respectively. In our data analysis, we discovered that the rate of malignancy (ROM) for category III (L3) was somewhat higher than the given value of 66.6%, with a comparison to 50% with a false positive rate of 7.5% [10,17,21]. The careful analysis of the cytology smear and the subsequent surgical specimen of these cases showed that two of these five cases had prominent follicular hyperplasia, and the smear showed many larger cells, which were reported as atypical cells. A similar observation was reported by Ankita Shibu Robert et al. in their cohort of cases [22]. In our opinion, flow cytometry is highly useful for reducing the occurrence of false positive cytology results by effectively demonstrating the characteristics of follicular hyperplasia through the strong expression of CD20 and CD10 by the population of interest. 

In this research, both the L4 (suspected to be malignant) and L5 (malignant) groups had very high ROMs of 63.6% and 88.7%, respectively. Interestingly, the L4 ROM that we observed in our sample was somewhat lower than what has been reported in several other investigations [10,17,19,23]. This is due to the use of ancillary methods like flow cytometry in their research, which was not performed in a good number of our cases in this category. A second diagnostic level based on supplementary procedures, as proposed in the categorization system, might further reduce the frequency of false negative results in this category.

Ancillary methods like as flow cytometry, immunocytochemistry (ICC), and molecular assays are crucial for providing a precise diagnosis, as well as for categorizing and subtyping lymph nodal aspirates [21,23]. We advocate for their utilization wherever possible. These tests have a crucial role in confirming diagnoses for category IV and V aspirates, while their relevance is restricted for categories I, II, and III. It is recommended to use these methods when dealing with patients that have questionable clinical characteristics, and the results should be analyzed along with clinical and cytomorphologic aspects. However, it is not possible to assess their role in our cohort of cases, as they were not utilized in all cases. It is essential to emphasize that our research has some more limitations. Firstly, the present research employed a retrospective methodology and had a somewhat limited sample size. Moreover, our institution is classified as a tertiary hospital in the region, which significantly influences the kind of patients we investigate. The majority of the cases sent to our hospital are for malignant conditions. This likely accounted for the increased number of cases in categories IV/L4 and V/L5.

Inspired by the transbronchial needle biopsy (TBNA) technique, Shaopeng Hua et al. [23] attempted to further investigate the utility of the fine-needle aspiration technique in lymph node pathology by modifying the conventional FNAC method and increasing the negative needle suction pressure. Through this modified method, a specialized needle was used, with the negative needle suction pressure raised to 20 mL and the needle thickness increased to 18 G. More cytology samples were obtained with this technique, and the results were comparable to those of the more conventional core needle biopsy (CNB). In the research group, 79.2% (38/48) of patients had a positive definitive pathological diagnosis, while in the control group, the rate was 82.5% (33/40) [23]. Based on their investigation, they found that modified needle aspiration biopsy is just as safe and accurate as CNB when it comes to diagnosis of superficial lymphadenopathy. All the fine-needle aspiration (FNA) samples in our study were obtained using the conventional approach, as our study is a retrospective one. Nevertheless, we are certain that evaluating this modified technique shows promise and paves the way for further future research. It will enhance the popularity of fine-needle aspiration (FNA) as a diagnostic method in clinical practice.

Despite the promising role of LN-FNA, the gold standard technique for a definitive diagnosis of a clinically suspicious lymphadenopathy remains lymph node tissue sampling, either through core needle biopsy or surgical excision. In a recent study by Antonio Facciorusso et al. [24], they were trying to explore the role of endoscopic ultrasound fine-needle aspiration (EUS-FNA) and endoscopic ultrasound fine-needle biopsy (EUS-FNB) in malignant cases of abdominal lymphadenopathy. They concluded in their study that EUS-FNB had a higher diagnostic accuracy and sensitivity than EUS-FNA. Both modalities had no adverse events. Thus, the results support the use of EUS-FNB for abdominal lymph node sampling.

## 5. Conclusions

In conclusion, when it comes to diagnosing LN aspirates, LN-FNAC provides a high degree of diagnostic accuracy. The suggested Sydney approach, if implemented, has the potential to aid in risk-stratification using cytology and to achieve consistency and repeatability in cytologic diagnosis. It seems to be a strong and promising method for reporting and categorization, but to determine its validity and reliability, bigger multi-centric research are needed.

## Figures and Tables

**Table 1:** The cytological characteristics of each group in the Sydney system for reporting lymph node cytology.

Category	The Cytomorphologic Features
L1: Inadequate/Insufficient	Scant cellularity; extensive necrosis; technical limitations that cannot be overcome.
L2: Benign	Suppurative and granulomatous inflammation; heterogeneous lymphoid population with small lymphocytes predominating, and often germinal centers with dendritic cells and tangible body macrophages.
L3: Atypical (cells) undetermined significance/atypical lymphoid (cells) of uncertain significance (ALUS/AUS)	Heterogeneous lymphoid population, features suggest a reactive process, follicular lymphoma cannot be excluded; excess of large cells (centroblasts or immunoblasts) or immature small lymphoid cells or cases where the atypical cells are not lymphoid cells.
L4: Suspicious	Small and/or medium-sized, monomorphic atypical lymphoid cells suspicious of lymphoma, but the cytomorphology alone is not sufficient; polymorphous lymphoid smears, few Hodgkin- or Reed–Sternberg-like cells are detected; large cell or Burkitt lymphomas scantly cellular; smears in which atypical cells suspicious for metastasis are detected, but are too scant to be diagnostic.
L5: Malignant	Non Hodgkin lymphoma(NHL); Hodgkin lymphoma (HL): appropriate cellular background and diagnostic Hodgkin and Reed–Sternberg cells; metastatic neoplasms.

**Table 2:** Distribution of patients according to specimen source, tissue correlation, Sydney system category, and final surgical specimen diagnosis (No.: 188) (No.: 188).

**Specimen source**	
Axillary LN	72 (38.3)
Cervical LN	20 (10.6)
Hilar mass	9 (4.8)
Inguinal LN	2 (1.1)
Neck LN	2 (1.1)
Paratracheal LN	18 (9.6)
Peripancreatic LN	1 (0.5)
Subcarinal LN	22 (11.7)
Submandibular LN	8 (4.3)
Supraclavicular LN	3 (1.6)
Thyroid	1 (0.5)
NA	30 (16)
**Tissue correlation**	
No	1 (0.5)
Yes	187 (99.5)
**Sydney system Category**	
Category I (L1): nondiagnostic/inadequate	6 (3.2)
Category II (L2): benign	49 (26.1)
Category III (L3): ALUS/AUS	15 (8)
Category IV (L4): suspicious	11 (5.9)
Category V (L5): malignant	107 (56.9)
**Final surgical specimen diagnosis**	
Benign	67 (35.6)
Malignant	121 (64.4)

N.B.: ALUS/AUS = atypical lymphoid cells of uncertain significance/atypical cells of undetermined significance.

**Table 3:** Validity and precision of the Sydney system category classification when compared to the final surgical specimen diagnosis.

Variable	Parameter	Estimate
Sydney system category classification results compared to the final surgical specimen diagnosis	True positive	95 (50.5)
	True negative	55 (29.3)
	False positive	12 (6.4)
	False negative	26 (13.8)
	Sensitivity	78.5%
	Specificity	82%
	Positive predictive value (PPV)	88.7%
	Negative predictive value (NPV)	67.9%
	Accuracy rate	79.7%

**Table 4:** Correlation between the final surgical specimen diagnosis and the Sydney system categories validity.

Cytologic Category as per the Proposed Sydney System for Reporting Lymph Node Cytopathology	Total	Final Surgical Specimen Diagnosis		χ2	*p*-Value
	No. (%)	Benign No. (%)	Malignant No. (%)		
L1: Inadequate/Insufficient	6 (3.2)	4 (6)	2 (1.7)	13.95	<0.001
L2: Benign	49 (26.1)	42 (62.7)	7 (5.8)		
L3: Atypical (Cells) Undetermined	15 (8)	5 (7.5)	10 (8.3)		
L4: Suspicious	11 (5.9)	4 (6)	7 (5.8)		
L5: Malignant	107 (56.9)	12 (17.9)	95 (78.5)		

**Table 5:** Risk of malignancy (ROM) associated with each cytologic diagnostic category of the proposed Sydney system for reporting lymph node cytopathology.

Cytologic Category as per the Proposed Sydney System for Reporting Lymph Node Cytopathology	Total No. of Cases with Histopathologic Diagnosis in Each Diagnostic Category (No.: 188)	Total No. of Cases Reported as Malignant on Histopathology (No.: 121)	Overall Risk of Malignancy (ROM) (%)
Category I (L1): Nondiagnostic/inadequate	6 (3.2)	2	33.3
Category II (L2): Benign	49 (26.1)	7	14.2
Category III (L3): ALUS/AUS	15 (8)	10	66.6
Category IV (L4): Suspicious	11 (5.9)	7	63.6
Category V (L5): Malignant	107 (56.9)	95	88.7

N.B.: ROM = risk of malignancy.

## Data Availability

Data are contained within the article.
